# Spatial transcriptomics profiling across tissue boundaries in the developing mouse brain

**DOI:** 10.1016/j.isci.2026.117765

**Published:** 2026-10-04

**Authors:** Vincent Hammer, Stephan Ossowski

**Affiliations:** 1 Institute for Medical Genetics and Applied Genomics, University of Tübingen, Tübingen, Germany; 2 Institute for Bioinformatics and Medical Informatics (IBMI), University of Tübingen, Tübingen, Germany

**Keywords:** tissue boundaries, gene expression, spatial transcriptomics, workflow, boundary distance, developmental biology, brain, meninges, expression patterns, neurodevelopment

## Abstract

Tissue boundaries preserve structural integrity and guide cell differentiation during development, despite constant stress from proliferation, cell death, and tissue deformation. To study gene regulation in these regions we developed REBOSEP, a workflow for analyzing spatial transcriptomics data to detect tissue boundaries and measure gene expression relative to boundary distance. REBOSEP uses hierarchical clustering to uncover shared regulatory patterns among genes, revealing highly diverse expression changes that can shift sharply across just a few cell layers.

Using this approach, the study generated the first resource of gene expression across tissue boundaries, covering five brain-meninges boundaries in the developing mouse brain. Analysis of the dorsal pallium-meninges boundary showed specific regulatory patterns in outer brain and meningeal layers, with strong links to developmental processes and distance-dependent regulation in major pathways, including those involved in neurodevelopmental disease and cancer. Overall, REBOSEP provides a systematic framework for studying gene expression regulation at tissue boundaries.

## Highlights

• REBOSEP reveals complex, non-linear expression changes across tissue boundaries
• Boundary aligned patterns reveal layer specific activity of key pathways
• Resource maps gene expression across five brain-meninges boundaries in mouse
• Clustering reveals genes sharing similar expression patterns across the boundary

## Graphical abstract

**Figure:**
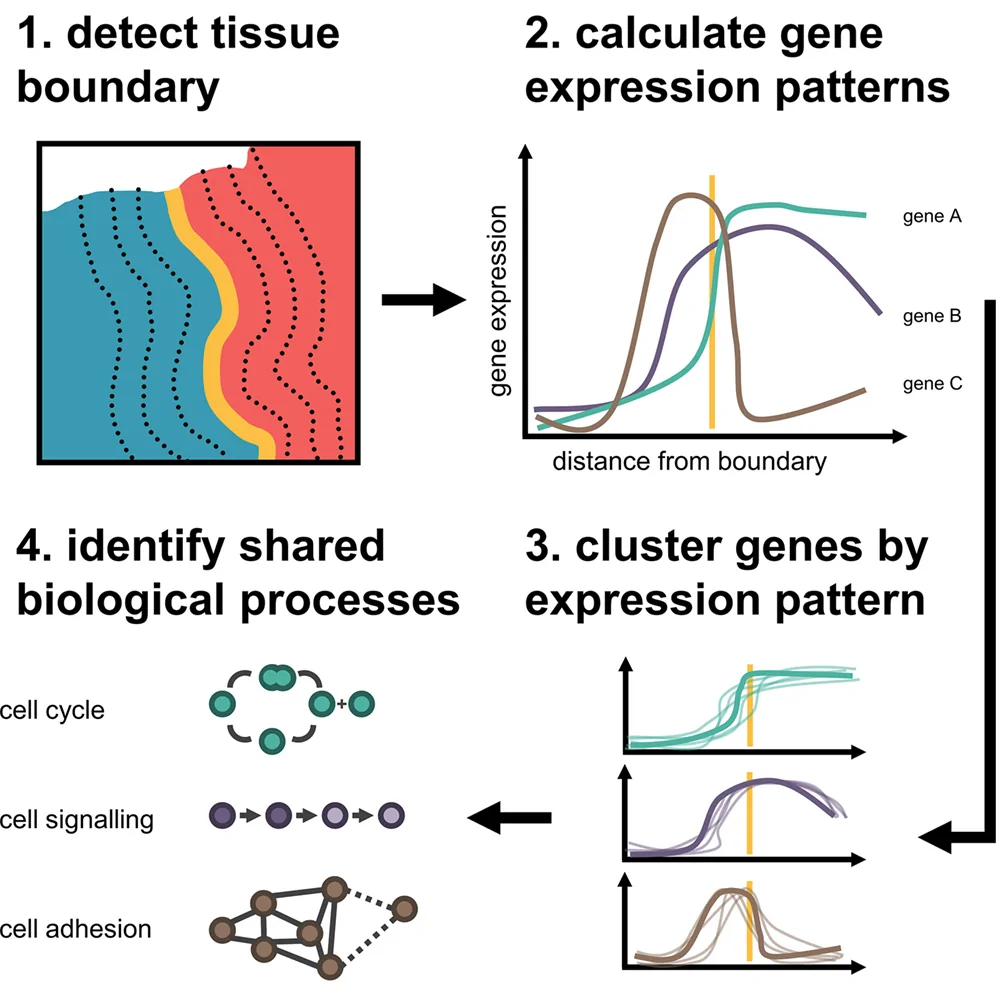

## Teaser

Biological sciences; Natural sciences; Neuroscience

## Introduction

Tissue boundaries play a crucial role in tissue growth and differentiation. The separation of functionally distinct cells is essential for proper development and maintenance of tissue structures, both at the boundary itself and in the surrounding regions. These boundaries are under constant stress from cell proliferation, cell death, and tissue deformation. Therefore, complex mechanisms have evolved for boundary formation and maintenance.^1^^,^^2^^,^^3^^,^^4^^,^^5^^,^^6^^,^^7^^,^^8^

Two main types of boundaries exist in undifferentiated tissue: compartment boundaries and non-lineage boundaries. In compartment boundaries cell identity is inherited and cell sorting preserves separation. Selector genes expressed within these compartments maintain homogeneity by preventing the intermixing of cells lacking the transcripts of these genes. In non-lineage boundaries, cell identity is determined by the cell’s neighborhood. A continuous signaling input is necessary for cell fate determination, which ultimately strengthens the separation between adjacent tissues.^2^^,^^4^

For maintenance of the established boundary, cell signaling is essential. Signaling pathways commonly associated with boundary maintenance are notch signaling and wingless signaling.^2^^,^^9^ In the dorsoventral compartment boundary of *Drosophila melanogaster* notch signaling maintains the boundary’s shape by locally strengthening the cell bonds.^10^^,^^11^ In early *Drosophila melanogaster* embryos Wnt signaling is required for myosin II enrichment at parasegmental boundaries. These actomyosin barriers maintain compartment boundaries through asymmetric cell adhesion.^12^ The importance of healthy tissue boundaries becomes especially apparent in cancer. One of the main hallmarks of tumor malignancy is the breakdown of the boundary between the tumor tissue and the healthy surrounding tissue.^13^^,^^14^ Deteriorating tissue boundaries are marked by boundary roughness, a known marker for invasiveness and worse prognosis in cancer.^15^^,^^16^ Accordingly, detection of boundary roughness in imaging data has been the focus of multiple studies attempting to predict malignancy.^17^^,^^18^^,^^19^ However, while tissue boundary roughness can indicate malignancy, several studies indicated that graded (more fuzzy) boundaries resulting from graded distributions of morphogens can also be functionally advantageous particularly during early development.^20^^,^^21^^,^^22^

Advancements in spatial transcriptomics have heightened the interest in studying localized transcriptional activity and cell-cell interactions. Latest technologies such as StereoSeq and VisiumHD even allow whole transcriptome sequencing at theoretically subcellular resolution.^23^^,^^24^ Leveraging these technologies, the tumor microenvironment and organogenesis in particular have become a focal point of research. Tools like BANKSY, BayesSpace, GraphST, Cell2location, and Tangram have been designed to identify tissue domains, offering improved annotation of tissues and detection of transcriptional microenvironments.^25^^,^^26^^,^^27^^,^^28^^,^^29^

The investigation of tissue boundaries using spatial omics data has recently been demonstrated in several studies. In 2023 Wu et al. published an analysis of the invasive zone in human liver, using Stereo-seq data.^30^ In this study, the authors segmented the boundary of the tumor and the healthy tissue into segments parallel to the boundary, and described the cell types, microenvironments, and metabolic reprogramming in these segments. CellNeighborEX, a method presented in 2023, detects interactions between neighboring tissues by identifying three segments: homotypic tissue A, homotypic tissue B, and heterotypic tissue (A + B) based on the spatial transcriptomes.^31^ Next, it detects differentially expressed genes between these regions to find neighbor-dependent gene expression (Kim et al., 2023).^31^ Spateo introduces a method that constructs internal coordinates even for complex tissue domains based on the boundary shape, and therefrom derives isolines and streamlines defining spatial sections.^32^ Spateo then detects graded or periodic gene expression patterns relative to these coordinates, where distances are defined in this internal potential-based coordinate system. The SPATA2 library implements distance-aware analyses using linear spatial trajectories or distance fronts and enables the detection of non-random spatial expression gradients.^33^ BELAYER models gene expression as piecewise linear functions of relative depth within tissue layers to jointly infer discrete layers and continuous variation within them,^34^ whereas GASTON and its extension GASTON-Mix use deep mixture-of-experts models to simultaneously learn spatial domains, one-dimensional internal coordinates, and domain-specific expression gradients.^35^^,^^36^ These methods provide powerful domain discovery and layer inference, but they are either conceptually and computationally heavier, focus on layer or domain segmentation, represent boundary-related expression in terms of piecewise structures, or they do not include unsupervised pattern identification.

In comparison, REBOSEP is an efficient, boundary-focused workflow that (i) accepts any existing tissue or domain annotation, (ii) represents gene expression as a function of physical distance to annotated boundaries, and (iii) detects shared, potentially complex expression patterns across genes at those boundaries. It operates downstream of any tissue annotation pipeline, using annotated regions to define and analyze tissue boundaries. Clustering of the detected gene expression patterns reveals complex, non-linear patterns shared by multiple genes, providing interpretable insights into gene regulation at tissue boundaries. We illustrate its potential with a resource of spatial gene expression patterns across five boundaries of the developing mouse brain, complementing domain- and layer-centric approaches.

Spatial transcriptomics data are even more affected by data sparsity than single-cell data. While technologies like StereoSeq and VisiumHD theoretically offer subcellular resolution, RNA capture rates necessitate spot binning, where n∗n spots are merged into bins.^23^^,^^24^ These then become the smallest units of analysis and define the achievable resolution. Despite this, data sparsity remains an issue for bin sizes at approximately cellular resolution. Additionally, these bins are formed without considering the position and identity of the cells covered.^37^ Cell segmentation methods attempt to address this issue, but suffer from high sparsity and poor cell detection. The data sparsity and the heterogeneous bins invariably lead to erroneous annotations.^38^

REBOSEP enables users to manage these challenges of boundary detection by integrating transcription of cells along extended boundary stretches. Additionally, it allows filtering based on annotation cluster size, boundary length, and interactive region of interest selection. It then reports the average expression of each gene along a cross section within user defined distance to the boundary. This presents gene expression as a continuous signal across tissue boundaries. Finally, users can cluster genes based on their expression patterns to identify shared regulatory patterns.

The development of the mammalian CNS is marked by its great complexity and vulnerability to dysregulation.^39^ Understanding the processes shaping embryogenesis in model organisms such as *Mus musculus* has given insights into the development of neurological disorders as well as cancer and provides avenues for the development of new treatments.^40^ To contribute to this effort, we applied REBOSEP to StereoSeq data of E16.5 mouse embryos. We analyzed five different boundary regions of the brain with the meninges to create an atlas, defined here as a comprehensive map of gene expression at brain boundaries, of gene expression patterns at boundary regions.

The dorsal pallium’s importance for the development of the neocortex, the role of the meninges during neurodevelopment, and their long continuous boundary, predisposed this region for an in-depth analysis.^41^^,^^42^ We found clear inhibition of proliferation, and enrichment of important developmental processes. We identified multiple pathways whose regulatory patterns are shaped by their proximity to the boundary. Investigation of known disease and cancer related genes revealed characteristic expression profiles across the cellular layers of different boundary region.

A cells activity is the product of its genetic programming, and surroundings. REBOSEP allows users to analyze this interplay at tissue boundaries, and presents gene expression in the context of its tissue. Gene expression across tissue boundaries is presented in its full complexity, allowing fast identification of regulatory patterns and similarities.

## Results

### A workflow to detect gene expression regulation across tissue boundaries

We designed REBOSEP to facilitate rapid identification of boundary specific gene expression patterns using spatial transcriptomics data. REBOSEP implements functionality for (i) preprocessing of the annotated spatial transcriptomics (ST) data, (ii) interactive region selection, (iii) boundary identification, (iv) generation and plotting of spatial gene expression patterns, (v) pattern clustering, (vi) data export, and (vii) exploratory analysis (Figure 1A).

**Figure 1:**
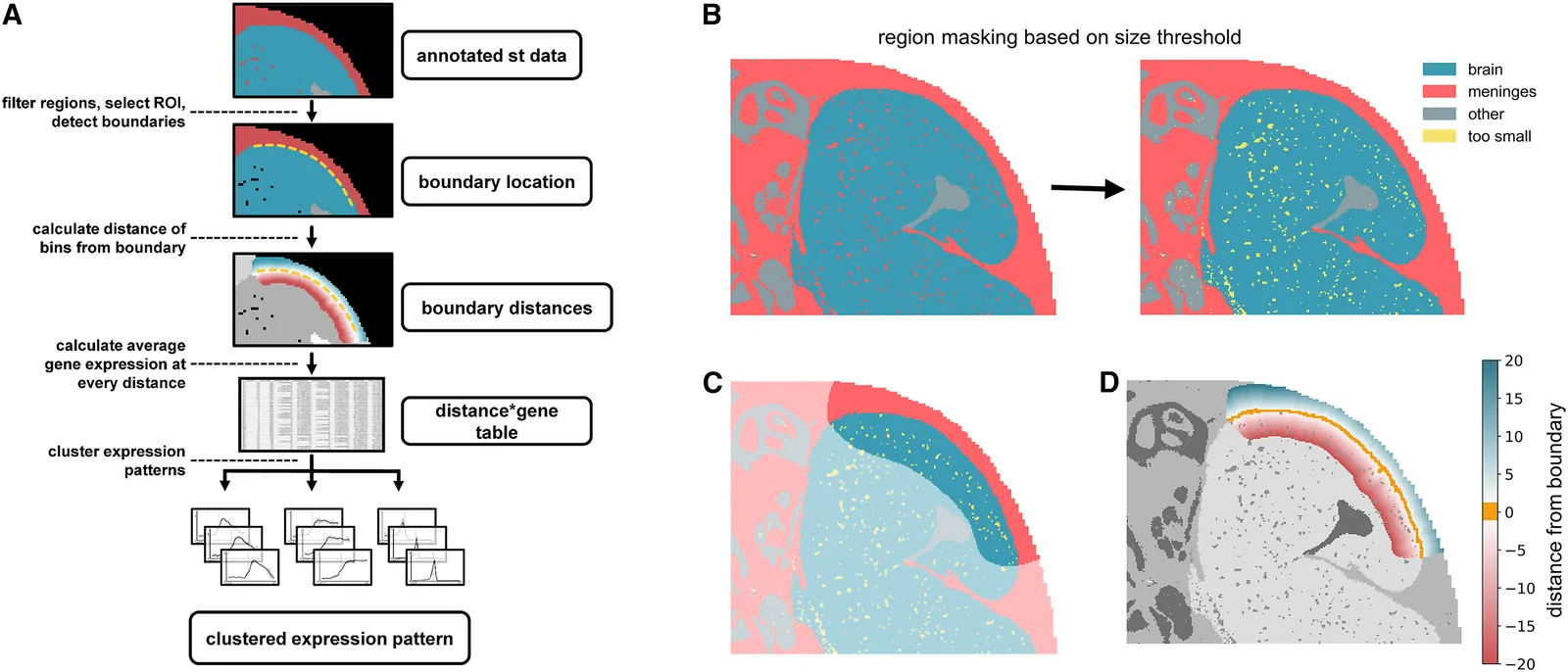
Overview of the workflow (A) Workflow overview. As input annotated spatial transcriptomics data are required. Using X, regions can be filtered and masked from boundary detection. After boundary detection, the distance of each bin from the boundary is calculated. The average expression of each gene at each distance from the boundary is then calculated. Next, the gene expression patterns can be clustered by similarity to identify clusters of genes sharing similar expression regulation across the boundary. (B) Before and after region filtering by size. Clustering of high resolution spatial transcriptomics data can result in inconsistent annotation in homogeneous tissues. Using REBOSEP, the result of clustering can be filtered by size of the region to remove minor clusters from boundary detection. (C) Interactive region selection for boundary detection. Boundaries are detected between user defined tissues in manually selected region, allowing the analysis of specific subsection of boundaries. (D) Final result of boundary distance detection, including highlight of the detected boundary.

As input, preprocessed and annotated spatial transcriptomics data from e.g., Stereo-Seq or Visium HD is required. The annotated regions can then be filtered by size (Figure 1B). Additionally, the exact boundary region of interest (ROI) can be manually selected (Figure 1C). The selection mask is stored in the data object and can be reapplied for reanalysis, guaranteeing reproducible results. For boundary detection, each bin that is adjacent to a bin with a different annotation is identified. To limit boundary discovery to boundaries between two tissues of interest, only bins annotated within a region of interest are considered (Figure 1D). These bins are then connected into continuous boundaries. Next, the minimum distance of all bins in the selected region from the boundary is computed (Figure 1D). Then, the average gene expression across the boundary, i.e., along the bin-sized distance intervals, is calculated. A table of these results (gene x distance) can then be exported.

Finally, the gene expression patterns can be clustered using signal clustering. Genes with similar expression patterns across the boundary are grouped together (Figure 1A). Genes with expression patterns of particular interest across multiple samples, e.g., pronounced overexpression only at the boundary, can be identified. Gene ontology (GO) enrichment analysis of a cluster can then generate quick insights into the major cellular processes linked to expression patterns. A detailed description of algorithms and statistical methods implemented in REBOSEP can be found in the STAR Methods section.

### Reliable detection of tissue boundaries despite noisy tissue annotations

Recent spatial transcriptomics technologies promise subcellular resolution, pointing to their minuscule spot size. Practically though, capture rates in these spots are too low for meaningful analysis, and so-called bins have to be formed. A bin *n* consists of *n*∗*n* spots, and summarizes all reads in these spots. Binning is done without consideration of cell position or identity. In StereoSeq for example the usually recommended bin size of 30–50 (approximately the size of 1–4 cells), generates some small, isolated bins annotated with random tissues in Leiden clustering (Figure 1B). The poor specificity of the annotation likely stems from the bad cluster separation in spatial data, as bins usually consist of multiple cells, sometimes even multiple cell types. Particularly the bins directly at the tissue boundary will contain transcripts from different cell types.

To detect long, continuous boundaries despite these issues, REBOSEP implements filtering functionality for the annotation and the detected boundaries. Small, random clusters can be masked from boundary detection (Figures 1B and 1C, highlighted bin). Additionally, the annotation of regions can be manually set. Because of diffusion during sample processing in the wet lab, gene expression will also be detected in regions not covered by tissue, i.e., cavities. These cavities will then be almost randomly assigned a cluster by most clustering algorithms. Manual annotation of individual clusters allows the user to fix such errors. Finally, the specific boundary region can be manually selected. This gives users the control to select specific subregions for boundary analysis. To avoid issues stemming from noisy Leiden annotation during boundary detection, REBOSEP can reconstruct long boundaries from smaller fragments, and bridge short gaps in boundaries.

### Profiling boundary specific gene expression regulation in the brain during mouse embryogenesis

From the publicly available MOSTA Stereo-Seq dataset, we selected sagittal sections from C57BL/6 mouse embryos.^23^ In each section, if applicable, we analyzed the following boundaries at a resolution of 30∗30 bins: dorsal pallium-meninges, midbrain-meninges, lower hindbrain-meninges, upper hindbrain-meninges, subpallium-meninges (Figure 2A). For simplification, we treated and refer to all tissue layers neighboring the brain as meninges. We detected tissue layers up to a depth of 300 μm into the brain. In the meninges, we observed a sharp drop in the number of bins detected more than 150 μm into the boundary in most regions, and therefore decided to limit distance detection to 150 μm (Figure S1).

**Figure 2:**
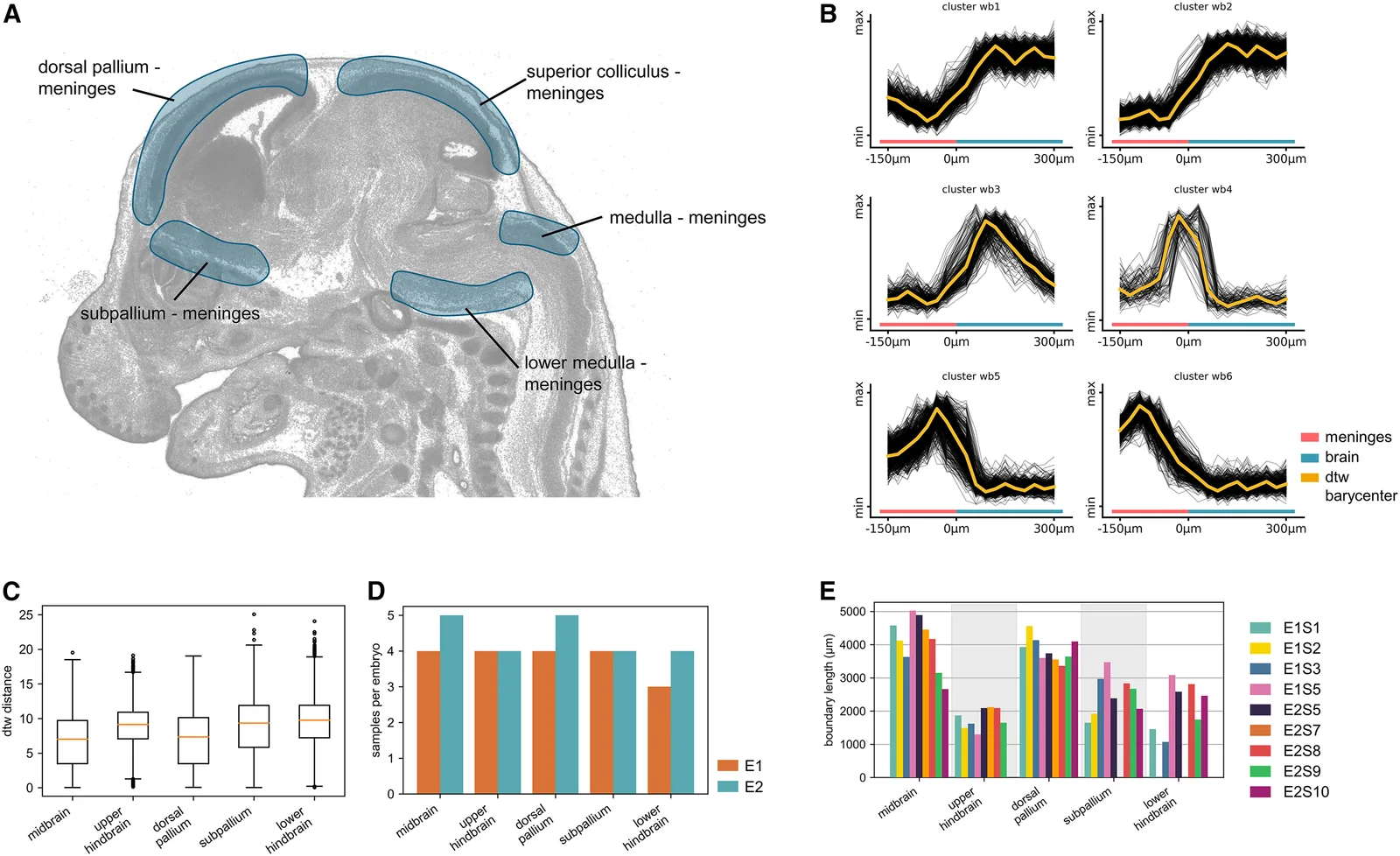
Analysis of 5 brain regions in the developing mouse brain (A) Analyzed regions in E16.5 *Mus musculus*, from the Allen Developing Mouse Brain Atlas. Allen Mouse Brain Atlas, mouse.brain-map.org. (B) Selection of expression patterns after hierarchical clustering. We selected clusters with clear enrichment at or near the boundary. Average pattern highlighted. (C) Average intrasample dtw distance of genes. (D) Number of samples for each analyzed boundary in embryo E1 and embryo E2. (E) Lengths of the analyzed boundaries. Expression levels are presented as *Z* score-transformed mean expression values.

To quantify the similarity between gene expression patterns, we used dynamic time warping (DTW), which aligns time series by locally stretching or compressing segments to minimize their overall distance. This approach can accommodate for minor pattern shifts between replicates that may arise from spot binning or RNA diffusion, while preserving the characteristic expression pattern. Using DTW with a restrictive warping window allowed us to differentiate patterns offset by as little as 30 μm, an important property particularly in genes whose expression is limited to only thin cellular layers.

Distribution of the intra-region DTW distances between replicates was comparable between regions, though we detected more outliers with decreasing bin counts (Figure 2C). Boundary lengths ranged from 1,740 μm to 3,667 μm, with most boundaries exceeding 2,000 μm (Figure 2C). We then performed hierarchical clustering of the gene expression patterns to find patterns shared by multiple genes. We selected 6 clusters (whole brain wb1–wb6) to highlight the considerable diversity of expression patterns (Figure 2B and Table S1). The detected expression patterns include localized overexpression near the boundary of varying degrees (clusters wb3, wb4, wb5), inhibition at the boundary (wb1), and constitutive expression in a tissue (wb3).

The data resource provided with this publication consists of the expression pattern of all genes captured by StereoSeq in each replicate. These expression patterns include lncRNAs and genes with yet unknown functions, such as Riken genes. Extending beyond the resource, REBOSEP can be applied to any boundary to investigate gene expression in any dataset generated by technologies comparable to Stereo-Seq or VisiumHD.

### Associating gene expression profiles with biological processes in the dorsal pallium—Meninges boundary

To demonstrate how REBOSEP can help understand the biological processes involved in boundary maintenance, we reapplied hierarchical clustering to the gene expression patterns at the dorsal pallium-meninges boundary (Figure 3A). We extended boundary detection up to 150 μm into the meninges, and 300 μm into the brain. This ensures that patterns spanning a wider cross-section along the boundary are detected, covering tissue depths that could not be analyzed in the joint analysis of all boundaries. We selected 6 expression patterns for enrichment analysis, including genes with constitutive activity in either meninges or the brain, and genes with strong enrichment in specific tissue layers (Figure 3C and Table S2). We applied GO enrichment analysis to identify the different biological processes associated with each of the expression patterns (Figure 3B and Table S3).

**Figure 3:**
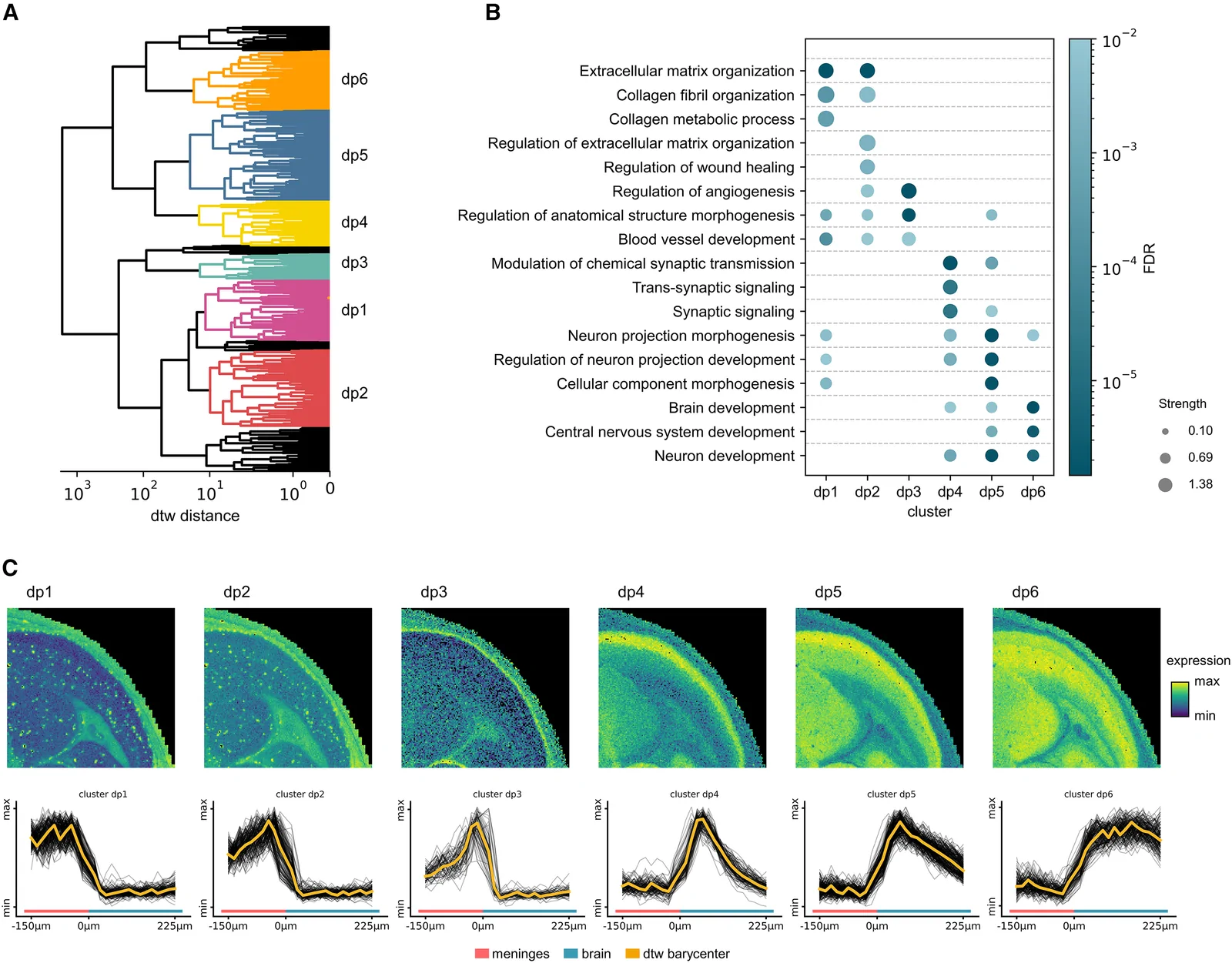
Analysis of gene expression regulation across the dorsal pallium-meninges boundary (A) Hierarchical clustering of the gene expression patterns at the dorsal pallium-meninges boundary. The clusters selected for further analysis (dp1 to dp6) are highlighted. (B) GO enrichment analysis of clusters dp1 to dp6. For each cluster, the top 3 biological processes were selected. (C) Heatmaps and line charts of the clusters dp1 to dp6. Heatmaps showing the dorsal pallium - meninges region. Heatmap expression levels are presented as log transformed mean expression values. Cluster pattern expression levels are presented as *Z* score-transformed mean expression values.

Genes with consistently high expression across all dorsal pallium layers (dp6) were overwhelmingly associated with developmental processes, including central nervous system, brain, and neuron development. Genes with consistently high expression in the meninges (dp1) were strongly enriched for extracellular matrix organization and processes directly linked to it. Genes showing overexpression in the meninges near the boundary with increased expression in all meninges layers (dp2) were greatly enriched for extracellular matrix organization. Additionally, we also detected enrichment of blood vessel development. We observed an even stronger enrichment of angiogenesis in dp3, a cluster consisting of genes with very strong enrichment in the meninges layers closest to the boundary. Cluster dp4, showing a similar pattern in the brain, was enriched for synaptic signaling and transmission. No enrichment for angiogenesis or extracellular matrix organization was detected. Cluster dp5 was strongly enriched near the brain boundary and was associated with neuron projection processes and neuron development. dp5 showed a more gradual decrease in expression toward deeper layers of the dorsal pallium, while dp4, in contrast, shows a sharp increase only very near to the boundary (Figure 3C).

In summary, clustering of gene expression patterns resulted in gene sets of great functional homogeneity. We identified specific cellular layers enriched for extracellular matrix organization, angiogenesis, and brain development. Gene clusters involved in angiogenesis, extracellular matrix organization, synapse development as well as synaptic transmission and signaling showed strongly localized peaky expression patterns in different cell layers close to the tissue boundary. Patterns associated with brain development, cellular matrix organization and neuron morphogenesis showed broader peaks and greater diversity in shapes and enriched processes.

### Robustness and validation of boundary-associated expression patterns

We evaluated how sensitive our clustering results are to the heuristic implementation of Leiden clustering (Figure S3) by repeating clustering 100 times with random seeds. Comparing clusters between runs we observed a vastly higher intra-cluster similarity compared to all other reported clusters. Even after randomly adding doubled noise patterns to expression patterns, intra-cluster distances remained small and at least eight times larger than inter-cluster distances (Figure S3). To assess how strongly our results depend on the choice of the upstream clustering algorithm, we performed the same robustness analysis again, but comparing Louvain with Leiden-derived clusters (Figure S4). Finally, we compared the gene expression patterns obtained from Leiden clustering with Louvain-derived gene expression patterns, by computing the pairwise DTW distance matrix between all genes using Spearman’s rank correlation (Figure S5). The resulting correlation was very high (Spearman ρ = 0.939, p << 1e−16), indicating that pairs of patterns that are close or distant under Leiden remain close or distant under Louvain.

Varying the spatial bin size had only a modest impact on the overall shape and alignment of cluster-averaged expression profiles, except at the coarsest resolution of 100 bins, where we observed a clear loss of spatial detail (Figure S6). Importantly, this bin size of 100 is already substantially larger than binning resolutions typically used in spatial transcriptomics analyses, indicating that boundary-associated patterns are robust across all practically relevant binning choices.

### Validation in an independent Visium HD dataset

We next asked whether the boundary-associated expression patterns inferred from the E16.5 Stereo-seq dataset are reproducible in an independent technology, specimen and embryonic stage. To this end, we analyzed E15 to E15.5 Visium HD datasets and extracted spatial expression profiles for the same gene sets defined by REBOSEP clusters. Visium HD-derived profiles closely resembled the Stereo-seq patterns, with similar positions of expression peaks, relative amplitudes, and rising or decaying trends (Figure S7). As expected from the slight developmental offset to the StereoSeq dataset, some clusters showed differences in peak sharpness and baseline levels, but overall pattern shapes were preserved. These observations indicate that the inferred boundary-associated expression patterns are reproducible across platforms and close developmental stages.

### Regulation of pathways at the dorsal pallium—Meninges boundary

Given the strong specificity of the enriched GO terms, we examined how selected pathways involved in boundary maintenance, cell proliferation, and cell-cell signaling are regulated at the dorsal pallium-meninges boundary. Distances are reported relative to the boundary, with positive values in the brain and negative in the meninges (Figure 4). For canonical Wnt signaling (mmu04310), we observed clear boundary-dependent modulation at both receptor and intracellular levels (Figure 4A). *Fzd1* and *Fzd2* were inhibited at the boundary, whereas Rspo3 was overexpressed around 75 μm, consistent with its role in promoting Wnt activity. Intracellular components *Apc*, *Ccnd2*, and *Tle4* showed strong patterns, with *Apc* peaking at -50 μm and *Ctnnd2* and *Tle4* displaying similar overexpression in the same layers. Notably, the downstream target *Ccnd2* was restricted to a narrow region around the boundary (−20 μm–20 μm), consistent with its role in cell-cycle regulation.

**Figure 4:**
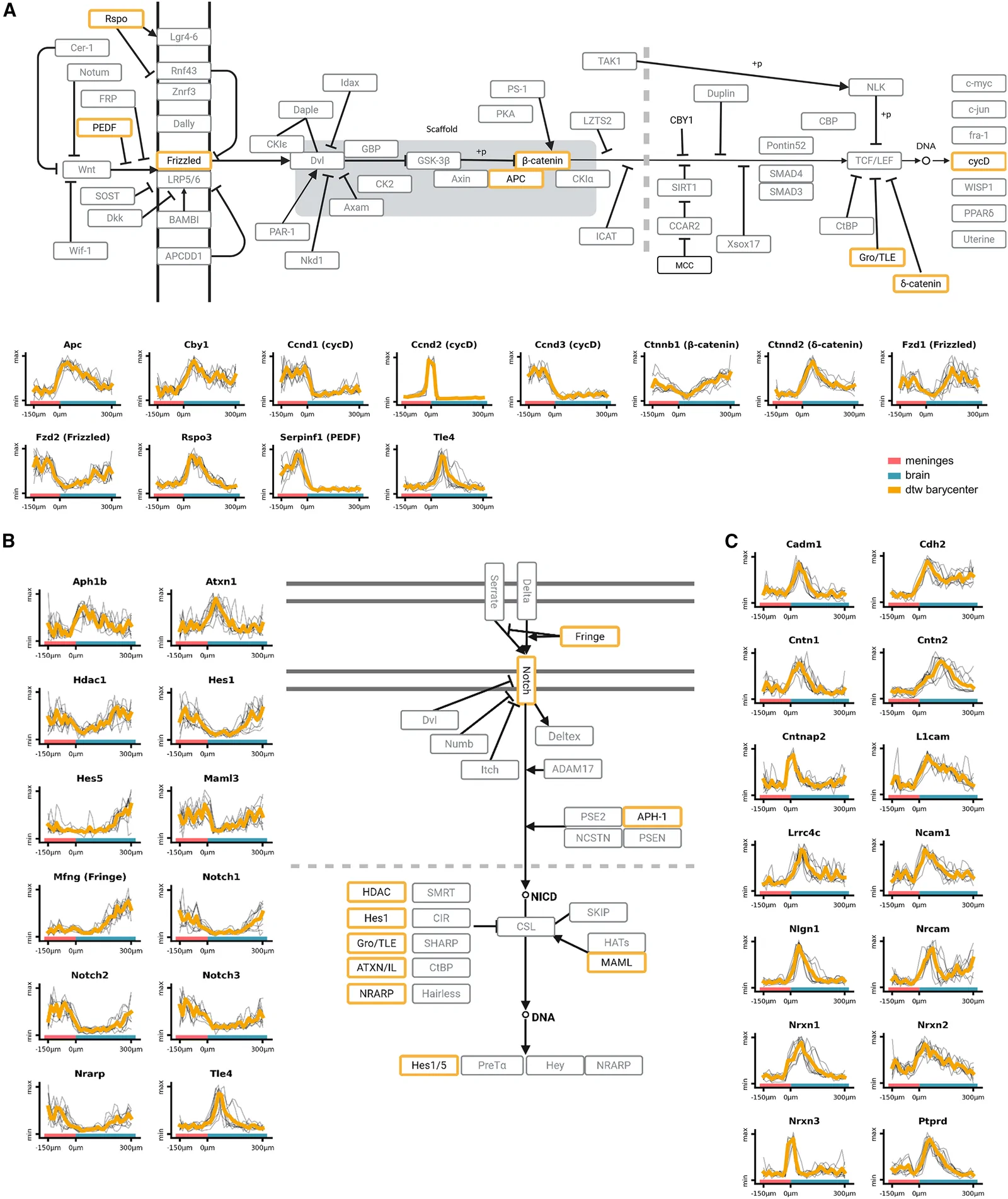
Expression patterns of a selection of genes involved in pathways across the dorsal pallium-meninges boundary (A) Canonical pathway of the Wnt signaling pathway (KEGG ID: mmu04310) (*n* = 9). (B) Notch signaling pathway (KEGG ID: mmu04330) (*n* = 9). (C) Neuron (presynaptic)-neuron (postsynaptic), and neuron (growth cone)-neuron (axon) interactions of the cell adhesion molecules pathway (KEGG ID: mmu04514) (*n* = 9). Gene expression levels are presented as *Z* score-transformed mean expression values.

Notch signaling (mmu04330), a short-range pathway essential for cell fate decisions and neurogenesis, showed broad inhibition in the brain side from ∼20 μm to 170 μm (Figure 4B). *Notch1*–*3* were highly expressed in the meninges but reduced at the boundary and in outer brain layers, accompanied by inhibition of downstream targets. *Hes5* was inhibited both in meninges and outer brain layers, while *Hes1* was increased in the meninges. Co-repressors such as *Hdac1* were inhibited in outer brain layers, whereas *Atxn1* and *Tle4* peaked around 30–75 μm; the activator *Maml3* was also inhibited in the outer brain. For differential adhesion, we analyzed the KEGG pathway mmu04514, which underlies brain morphogenesis, learning and neural migration (Figures 4C and S2). Genes mediating neuron-neuron and growth cone-axon interactions showed expression largely confined to a thin boundary region (∼50–150 μm) with low expression in the meninges. Multiple genes, including *Cntnap2*, *L1cam*, *Ncam1*, *Nrxn1*, *Nrxn2*, and *Ptprd*, displayed a gradual decrease toward deeper brain layers.

Taken together, pathway-level visualization with boundary-aligned gene expression revealed not only which processes are active at this border, but also how they are spatially regulated: notch signaling is broadly inhibited in outer brain layers, adhesion molecules are tightly confined to a ∼100 μm band in the brain, and Wnt signaling components show highly localized overexpression in both meninges and adjacent brain tissue with concurrent depletion of other core elements in the same region.

## Discussion

Maintenance of cell boundaries is crucial for the development of healthy tissues.^2^ The consequences of misregulation during embryogenesis are extensive; from structural developmental anomalies to malignant growths in adult tissues.^43^^,^^44^

The introduction of modern spatial transcriptomics has opened the door to a deeper understanding of tissue boundaries. Untargeted methods like StereoSeq or whole transcriptome probe-based methods like VisiumHD, allow researchers to discover and investigate genes involved in boundary maintenance and pathogenesis at a far greater pace and lower cost than ever before. Moreover, working without *a priori* assumptions on the involved genes promotes the discovery of new regulatory mechanisms and gene functions. To take advantage of these new technologies and their potential for the research of boundary regions, we developed REBOSEP. Using REBOSEP, boundaries can be quickly identified and the gene expression patterns across the boundary detected. Finally, hierarchical clustering of these patterns reveals regulatory trends across the boundary and GO analysis reveals essential functions of clusters.

Compared to existing tools for boundary analysis in ST data, our approach combines the following advantages: (1) detection of continuous gene expression signals, (2) built-in functionality for noisy tissue handling, (3) interactive region selection, (4) identification of common expression patterns with no *a priori* assumptions needed, and (5) intuitive distance metrics. REBOSEP does not simplify the boundary region into broad categories. Instead, the continuous changes of gene expression in a cross-section of up to 300 μm from a boundary are detected and reported. It is applicable even in noisy ST data and complex tissue structures thanks to region and boundary filtering capabilities. Interactive region selection allows the user to focus on the exact region of interest. Finally, the hierarchical clustering of the gene expression patterns means no assumptions have to be made on gene expression, and allows the user to find any regulatory pattern shared by genes.

We applied REBOSEP to five boundary regions in the developing mouse brain and made the gene expression patterns publicly available. We expect this resource to offer great potential for a wide range of research questions. It offers new insights into healthy embryogenesis and pathogenesis in one of the most complex tissues. Genes with unknown functions, many lncRNA genes for example, are now annotated with a boundary-specific expression pattern providing clues to their function. Genes active in these regions can now be precisely localized in the brain during the final stages of embryogenesis.

In the dorsal pallium REBOSEP helped us to quickly identify the underlying biological processes activated and suppressed at its boundary with the meninges. We found expression patterns with strong enrichment for important developmental processes of the brain, enrichment for processes associated with the blood brain barrier, and modulation of synaptic activity. Visualization of pathways involved in boundary maintenance helped understand their regulation and the activity of the individual components. Finally, we observed clear patterns of boundary mediated expression regulation in key oncogenes and developmental disorder-associated genes.

### Boundary-specific expression profiles of genes and pathways associated to cancer and developmental disorders

Boundary-specific analysis of Wnt signaling and cell adhesion pathways revealed several core components with strong enrichment at tissue borders, including *Apc*, *Ccnd2*, *Tle4*, and *Ctnnd2*, as well as the adhesion molecules *L1cam*, *Nrcam*, *Cadm1*, and *Cntn1*. Many of these genes are known to promote or restrain tumor cell proliferation, invasion and metastasis in a context-dependent manner, suggesting that genes with strong boundary-specific expression in development are promising candidates for studying how tissue borders are remodeled or co-opted in cancer.^45^^,^^46^^,^^47^^,^^48^^,^^49^^,^^50^^,^^51^^,^^52^^,^^53^^,^^54^^,^^55^^,^^56^

Genes overexpressed at the boundary in cell adhesion pathways were also strongly linked to developmental disorders, including ASD-associated genes (*Cadm1*, *Lrrc4*, *Nlgn1*, *Nrcam*, *Nrxn2*) and genes implicated in neuronal migration and broader developmental syndromes (*Cdh2*, *Cntn1*, *L1cam*, *Lrrc4b*, *Ptprd*). The involvement of these genes in both cancer biology and neurodevelopment underscores the value of spatial boundary-specific profiles for pinpointing candidate mechanisms and potential therapeutic targets.^57^^,^^58^^,^^59^^,^^60^^,^^61^^,^^62^^,^^63^^,^^64^^,^^65^^,^^66^

The significance of these genes in both cancer and developmental biology further highlights the potential of spatial boundary-specific gene expression profiles for identifying candidate mechanisms and therapeutic targets. Genes with unknown or poorly characterized functions, including many Riken genes, emerge as particularly promising candidates. By applying REBOSEP to the dorsal pallium-meninges boundary, we generated a resource cataloging boundary-aligned expression patterns of thousands of genes in healthy embryonic tissue, highlighting candidates that may contribute to boundary maintenance in development and whose perturbation can now be tested in dedicated disease models.

### Limitations of the study

Our gene-level interpretations are exploratory and hypothesis-generating. Validation of individual candidate genes and pathways will require additional spatial datasets and/or targeted experimental studies, which are beyond the scope of this work.

The main limitation for identifying gene expression patterns is the quality of the ST data and its dependence on upstream clustering. Inaccurate tissue segmentation propagates into boundary definitions and the patterns inferred by REBOSEP. In our datasets, REBOSEP already yields robust and interpretable patterns with basic single-modality Leiden clustering, but more complex tissues, heterogeneous tumors, or subtle boundaries may require more advanced, multimodal domain-calling strategies that integrate histology and ST. REBOSEP can directly build on such improved, image-informed segmentations.

Current capture rates and data quality also limit spatial resolution and the number of genes that can be analyzed. True single-cell resolution is not yet achieved, and binning reduces spatial precision and blurs boundaries. Low capture rates in untargeted ST further require bin sizes larger than a typical cell and restrict REBOSEP to longer boundaries to obtain meaningful expression patterns.

All publicly available datasets analyzed in this study were derived from female mice; therefore, we could not assess sex-specific effects or include male animals. While the studied biological processes are not expected to be strongly sex-dependent, the generalizability of our findings to male mice requires confirmation in sex-balanced datasets.

## Resource availability

### Lead contact

Requests for further information and resources should be directed to and will be fulfilled by the lead contact, Stephan Ossowski (stephan.ossowski@med.uni-tuebingen.de).

### Materials availability

This study did not generate new unique reagents.

### Data and code availability

• The StereoSeq data are available under project ID CNGBdb: CNP0001543 from CNGBdb.
• The VisiumHD datasets of mouse embryos are available on the 10× Genomics website, listed under the IDs Visium HD Spatial Gene Expression Library, Mouse Embryo and Visium HD 3′ Gene Expression Library, Mouse Embryo (Fresh Frozen).
• The gene expression patterns of all analyzed regions have been deposited at https://github.com/imgag/REBOSEP_resource and are publicly available as of the date of publication.
• All original code has been deposited at https://github.com/imgag/REBOSEP and https://github.com/imgag/REBOSEP_resource and is publicly available as of the date of publication.
• Any additional information, including region masks required to reproduce the results reported in this study, is available at https://github.com/imgag/REBOSEP_resource.

## Acknowledgments

V.H. and S.O. were supported by the 10.13039/501100000780European Union via the project European Rare Disease Research Alliance (ERDERA), GA n°101156595, funded under call HORIZON-HLTH-2023-DISEASE-07 and the 10.13039/501100007601European Union’s Horizon 2020 research and innovation program under grant agreement no 779257 (Solve-RD). S.O. received funding from the German Research Foundation (DFG) under grant numbers 514060894, 514208594, and 514177729.

V.H. received funding from the German Research Foundation (DFG) under grant number OS 647/1-1 AOBJ: 676259.

V.H. and S.O. received funding from the German Research Foundation (DFG) as part of the German Human Genome-Phenome Archive (GHGA) under grant number 441914366.

## Author contributions

V.H. analyzed data and contributed to manuscript writing. S.O. supervised the study and manuscript writing. All authors reviewed and approved the final manuscript.

## Declaration of interests

The authors declare no competing interests.

## Declaration of generative AI and AI-assisted technologies in the manuscript preparation process

During the preparation of this work the authors used Perplexity in order to improve the readability and language of the manuscript. After using this tool/service, the authors reviewed and edited the content as needed and take full responsibility for the content of the published article.

## STAR★Methods

### Key resources table

**Table:** 

REAGENT or RESOURCE	SOURCE	IDENTIFIER
**Deposited data**		

StereoSeq datasets of mouse embryos	https://db.cngb.org/^67^	project ID: - CNP0001543 sample IDs: - CNS0520797 - CNS0520799 - CNS0520798 - CNS0520800 - CNS0520810 - CNS0520812 - CNS0520816 - CNS0520817 - CNS0520818
VisiumHD datasets of mouse embryos	https://www.10xgenomics.com/datasets^68^	- Visium HD Spatial Gene Expression Library, Mouse Embryo - Visium HD 3' Gene Expression Library, Mouse Embryo (Fresh Frozen)
REBOSEP resource	This study	https://github.com/imgag/REBOSEP_resource

**Software and algorithms**		

SAW	https://github.com/STOmics/SAW	V6.0.0
StringDB	https://string-db.org/	V12.0
SciPy	https://github.com/scipy/scipy	V1.10.1
StereoPy	https://github.com/STOmics/stereopy	V1.0.0
REBOSEP	This study	NA
tslearn	https://github.com/tslearn-team/tslearn	V0.6.3

### Experimental model and study participant details

We obtained raw StereoSeq data of E16.5, C57BL/6 mice, from the China National GeneBank Database (CNGBdb), project ID CNP0001543 (sample IDs CNS0520797, CNS0520799, CNS0520798, CNS0520800, CNS0520810, CNS0520812, CNS0520816, CNS0520817, CNS0520818), and the corresponding CID to barcode files from the STomicsDB MOSTA database. All original work was conducted under the approval of the Animal Care and Use committee of the Guangzhou Institutes of Biomedicine and Health, Chinese Academy of Sciences (license number IACUC2021002).

We obtained processed VisiumHD data from the official 10X Genomics datasets repository. We selected both “Visium HD Spatial Gene Expression Library, Mouse Embryo “samples of female E15.5 embryos. Additionally we included the „Visium HD 3′ Gene Expression Library, Mouse Embryo (Fresh Frozen)” sample of a female E15 mouse embryo. The datasets are available under a Creative Commons Attribution 4.0 International license. Ethical oversight and animal-use approvals for the original tissue collection were not reported in the publicly available dataset documentation; therefore, no approval number can be provided here.

### Method details

#### Preprocessing of mouse embryo samples

We selected the StereoSeq samples based on their complete and undamaged coverage of the regions of interest, and removed damaged samples from the analysis. We processed the StereoSeq data using the SAW workflow v6.0.0 with default parameters. The genome index file for the mouse reference genome GRCm39 was built using *singularity exec SAW_v6.0.0.sif mapping* with default parameters.

Basic preprocessing of the StereoSeq samples was performed using StereoPy v1.0.0. During data loading (function *st.io.read_gef*), bin size was set to 30 via *bin_size*, and the region selected using *region*. We removed low quality bins using *tl.filter_cells*. We excluded bins with < 100 reads, < 50 genes, and >= 7% mitochondrial content. (*min_gene*, *min_n_genes_by_counts*, *pct_counts_mt*). We selected the VisiumHD samples at a resolution of 8μm.

For annotation, we used the marker genes provided by the MOSTA database (https://ftp.cngb.org/pub/SciRAID/stomics/STDS0000058/Table/Markers_used_for_annotations_in_the_MOSTA.xlsx). With the StereoSeq samples e performed sctransform normalization (*tl.sctransform* with *n_genes=2000*), PCA (*tl.pca* with *use_highly_genes=False*, *n_pcs=30*), computation of a neighborhood graph (*tl.neighbors* with *pca_res_key='pca'*, *n_pcs=30*), and UMAP projection using the neighborhood graph (*neighbors_res_key='neighbors'*). Resolution of the Leiden clustering (*tl.leiden*, parameter *resolution*) was set on a per-sample basis to identify all tissues of interest while limiting overclustering. Finally, the data was converted into anndata format using *st.io.stereo_to_anndata* (with *flavor=“scanpy”*), and normalized using *sc.pp.normalize_total* with *target_sum=1e4*. For the VisiumHD samples we used the precomputed kmeans clustering results.

#### Functionality implemented in REBOSEP

The REBOSEP workflow consists of a set of functions designed for sample preprocessing, region selection, boundary detection, calculation of expression profiles, pattern clustering, as well as data export and visualization. The function *filter_regions* allows users to filter bin annotations by calculating the size of each contiguous subregion formed by bins sharing the same annotation. This preprocessing reduces noise and prevents detection of spurious boundaries. Additionally, the detection of these contiguous subregions simplifies data handling, as the function *set_annotation_by_region* allows the annotation of bins by their affiliation with these subregions. Specific regions can be selected using the function *select_region_of_interest_categorical*, which starts an interactive freehand selection tool. The function *find_boundary_distances detects* the distance of bins from the boundaries between two user defined tissue annotations. Masked regions (created with the function *select_region_of_interest_categorical*), minimum boundary length, boundary width, and gap size in boundaries can be set. Calculation of the average gene expression levels and data export are handled by the function *create_dist_gene_table*. A Jupyter notebook demonstrating a standard REBOSEP analysis and the analyses of all samples in this paper can be found at https://github.com/imgag/REBOSEP.

#### Analysis of tissue boundaries with REBOSEP

##### Annotation preprocessing

To remove small, spurious clusters from the annotated StereoSeq data, we filtered the annotation using the function *filter_regions*, which first detects all contiguous regions (i.e. all bins with same annotation, in direct contact), and then calculates the size (in bins) of each region. Then, all regions smaller than the threshold, set with parameter *minimum_region_size,* can be masked from the analysis. We set the parameter *minimum_region_size* specifically to each sample, aiming to remove all small, irrelevant clusters.

##### Boundary detection

We then detected the boundaries between the brain and the meninges using the function *find_boundary_distances*. During boundary detection using *find_boundary_distances*, all bins neighboring a differently annotated tissue are detected, unless masked by the *excluded_clusters* parameter. These are referred to as boundary bins. Next, each unique boundary is detected by recursively linking all boundary bins that are within the range defined by *boundary_range* of each other. The *boundary_range* parameter defines the radius of the search space for each boundary bin, within which boundary bins are considered as belonging to the same boundary. The shape of the search space is defined by *skimage.draw.disk*, with the *shape* parameter being set to *boundary_range*. This allows identifying long continuous boundaries despite short interruptions, whether from *filter_regions*, or damage from sample handling for example. Once all boundary bins have been assigned to boundaries, the boundaries can be filtered by length, using the *min_boundary_length* parameter. We set the minimum boundary length (parameter *min_boundary_length*) custom for each sample, usually ∼400 bins. The boundary range (parameter *boundary_dist*) was set to 1 for all samples.

##### Distance calculation

Next, the *find_boundary_distances* function calculates the distance of non-boundary bins from the detected boundaries. All boundary bins are used as centers for *skimage.draw.disk* shapes with a radius set by parameter *max_dist*. All bins covered by this shape are annotated with the value of *max_dist*. Then, the radius of the *skimage.draw.disk* space is iteratively decreased. Each time, all bins covered are annotated with the new, smaller radius value. This ensures that bins are always annotated with their smallest distance to a detected boundary. We limited the maximum distance from the boundary to 20 bins during the analysis of all samples.

##### Manual region selection

We then manually selected the boundary regions of interest using the function *select_region_of_interest_categorical*, aiming for long continuous boundaries, in undamaged tissue. Using *select_region_of_interest_categorical*, a specific subregion can be selected interactively. This allows the user to mask tissue sections from the analysis. During sample processing, we excluded regions where multiple boundaries were in close proximity (<= 20 bins) as well as damaged tissue, to avoid confounding gene expression patterns.

##### Data export

We exported the expression patterns as tsv files using the function *create_dist_gene_table* (with parameter *raw_counts_layer* set to the layer containing the raw read counts). *create_dist_gene_table* calculates the average expression of each gene at each distance (in bins) from the boundary, that is the expression pattern. Additionally, the total number of bins at each distance is reported.

We saved the anndata objects containing all intermediate results and the final analysis results using *anndata.write* (v0.9.2).

#### Filtering of gene expression patterns

We filtered the gene expression patterns before hierarchical clustering based on two criteria: sparsity and similarity across replicates. To filter by sparsity, we removed expression patterns where in at least half of the distances no expression had been detected, as these gene patterns were too inconsistent across replicates.

To filter genes by similarity, we used dynamic time warping (DTW). DTW alignment seeks to minimize Euclidean distance between time series, allowing for shifts in the data. This pairs nicely with the gene expression patterns of up to 40 bins observed across boundaries, as slight inaccuracies (∼1 bin) are to be expected between detected boundaries. First, we applied zscore normalization and a Savitzky-Golay-Filter for smoothing (using *SciPy v1.10.1 zscore* and *SciPy v1.10.1 savgol_filter* with *window_length=5*, *polyorder=2*). Then, we calculated the average pairwise distance using *aeon v0.7.1 dtw_pairwise_distance* with the *window* parameter set to mimic a Sakoe-Chiba radius of 2, and removed all genes with an average pairwise distance over 6.

We chose a restrictive Sakoe-Chiba window allowing for minimal warping to avoid over-alignment of distinct patterns. Because some bins inevitably lie directly on the tissue interface and contain mixtures of both adjacent tissues, the inferred boundary position is intrinsically uncertain. A Sakoe-Chiba radius of 2 for the pairwise distances addresses these inaccuracies without smoothing over bigger shifts between patterns.

#### Hierarchical signal clustering

To find genes sharing similar expression patterns, we used hierarchical clustering. First, we calculated the average gene expression pattern for each gene from all replicates using dynamic time warping barycenter averaging (*tslearn.barycenters* with *sakoe_chiba_radius=1* to allow for series offset). Dynamic Time Warping Barycenter Averaging (DBA) is a method for computing the average of a set of time series by aligning them using Dynamic Time Warping (DTW). The average is then iteratively updated to minimize the overall DTW distance. This preserves the characteristic expression pattern, despite slight positional offsets or inaccuracies.

We then clustered these average patterns using the *SciPy* implementation of Ward’s linkage. Ward's linkage is a hierarchical clustering method that merges clusters so as to minimize the total cluster variance, by iteratively joining the pair of clusters that results in the smallest total of squared distances once joined. For visualization we picked clusters with strong spatial patterns, i.e. clear inhibition or overexpression close to the boundary.

#### Clustering robustness analysis

Detailed documentation of the robustness analysis is provided in the supplementary data (Methods S1–S3).

#### Spatial expression pattern visualization

##### Heatmaps

To generate heatmaps of a set of genes in a tissue (such as Figure 3C), we normalized read counts using the StereoPy function *tl.normalize_total* with *target_sum=10000*. Then, we transformed the read counts logarithmically using the *StereoPy v1.0.0* function *tl.log1p*. Next, we summed up the expression of all genes in the set in each bin. For color scaling, we applied linear color scale normalization.

##### Gene expression patterns

To visualize the time-series-like gene expression patterns across a boundary, we calculated the DTW barycenter of the gene sets using *dtw_barycenter_averaging* from *tslearn v0.6.3*, using *sakoe_chiba_radius=1* (see Method S4). The expression curves were plotted using the Matplotlib function *plt.plot*.

##### Enrichment analysis

Enrichment analysis is used to find statistically overrepresented biological categories (such as pathways, Gene Ontology terms, or other gene sets) in a given set of genes. We used StringDB v12.0 0 (https://string-db.org/). Active interaction sources were set to experiments and databases only. Minimum required interaction score was set to highest confidence (0.9). All other settings were left as default. The full results are included in the supplementary data.

##### Pathway analysis

We selected the pathways mmu04310, mmu04330, and mmu04514 from the KEGG database. To filter out genes with low pattern similarity across replicates, we calculated for each gene the average pairwise distance across replicates using *tslearn dtw_distance* with *sakoe_chiba_radius = 1*. We removed all genes with an average pairwise distance over 20. Out of the genes associated with these pathways, we chose to visualize genes with clear signs of overexpression or inhibition near the boundary.

### Quantification and statistical analysis

No new biological samples were collected, and no sample-size calculation, randomization, or blinding was performed because this study involved secondary analysis of publicly available spatial-transcriptomic datasets. The unit of analysis was bin 30, unless specifically clarified otherwise.

Gene-expression profiles were quantified as the mean expression of each gene among spatial bins at each integer distance from a detected tissue boundary.

DTW distances were used as descriptive similarity measures and for prespecified filtering and clustering; no *p* values were calculated for DTW distances, DTW barycenter averaging, or hierarchical clustering. Thresholds for gene-pattern inclusion were an average pairwise DTW distance of 6 for the clustering analysis and 20 for the pathway visualization, as described in method details.

STRING v12.0 functional-enrichment analysis was performed as described in method details. Significance was determined using STRING-reported false-discovery-rate-adjusted *p* values, with an FDR threshold of 0.05. Full enrichment results, including adjusted ppp values, are provided in the supplemental information section.

Statistical hypothesis tests were not performed unless explicitly stated otherwise.

## Supplemental information

Document S1. Figures S1–S7, Table S1, and Methods S1–S4

Table S2. dp_enrichment_all.xlsx: STRING-db analysis of dp clusters, related to Figure 3B

Table S3. dp_clusters_to_genes.xlsx: list of the genes in each dp cluster, related to Figure 2B
