# Supplementary material for: Spatial transcriptomics profiling across tissue boundaries in the developing mouse brain
Source: iScience. 2026 Oct 4;29(11):117765. doi: 10.1016/j.isci.2026.117765 (PMC13659672; doi:10.1016/j.isci.2026.117765)
Supplement: Supplementary file 1 [file mmc1.pdf]

iScience, Volume *29*

Supplemental information

Spatial transcriptomics profiling across tissue boundaries in the developing mouse brain

Vincent Hammer and Stephan Ossowski

Supplementary Figure 1.

A

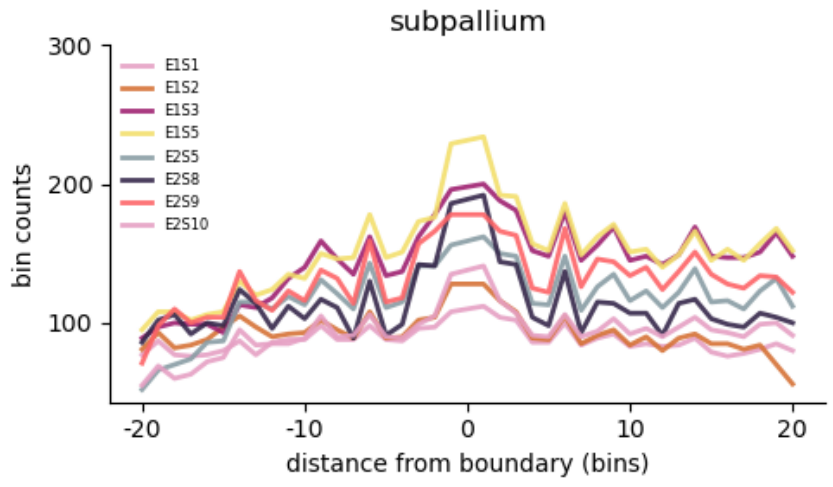

B

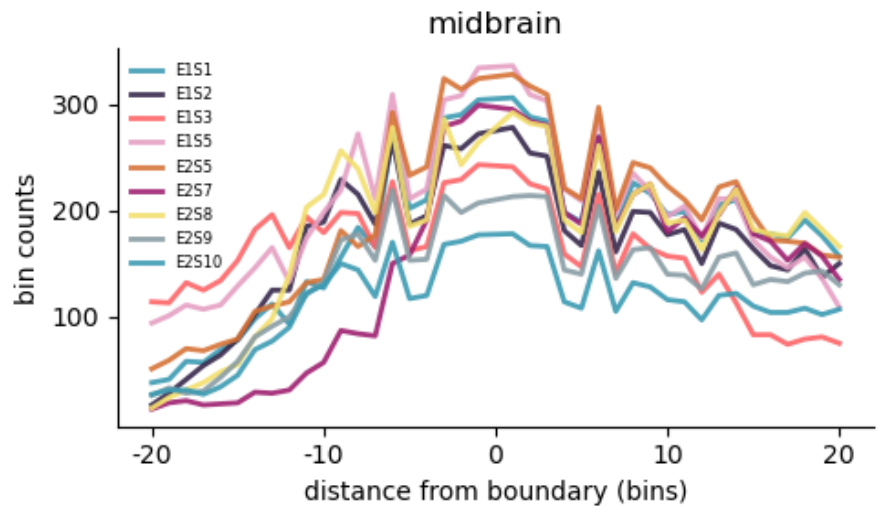

C

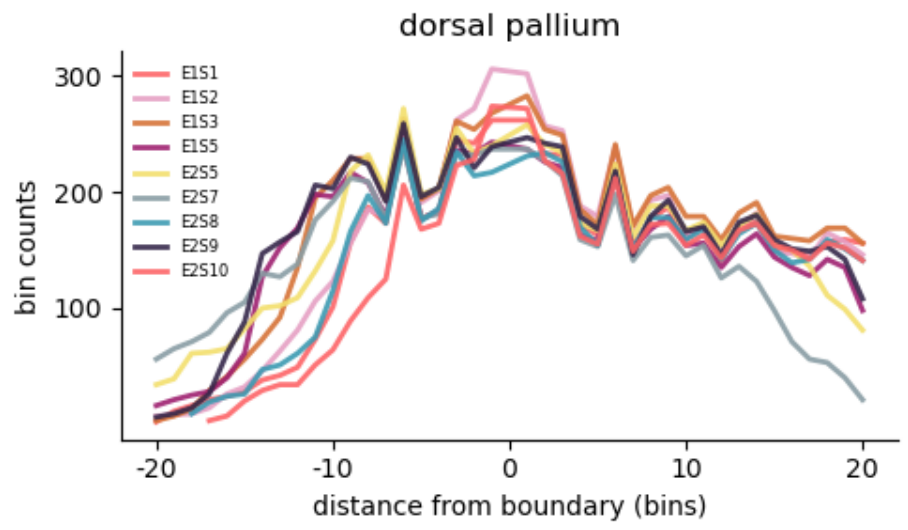

D

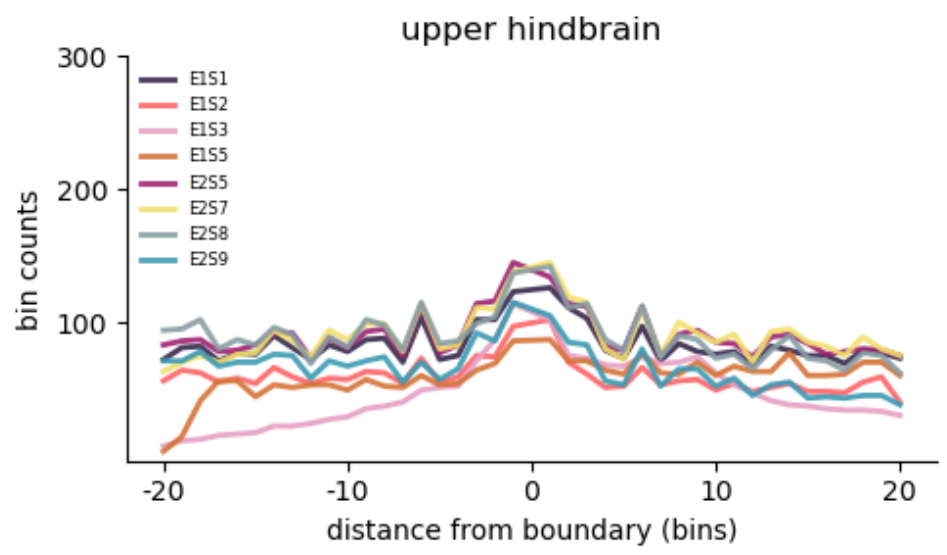

E

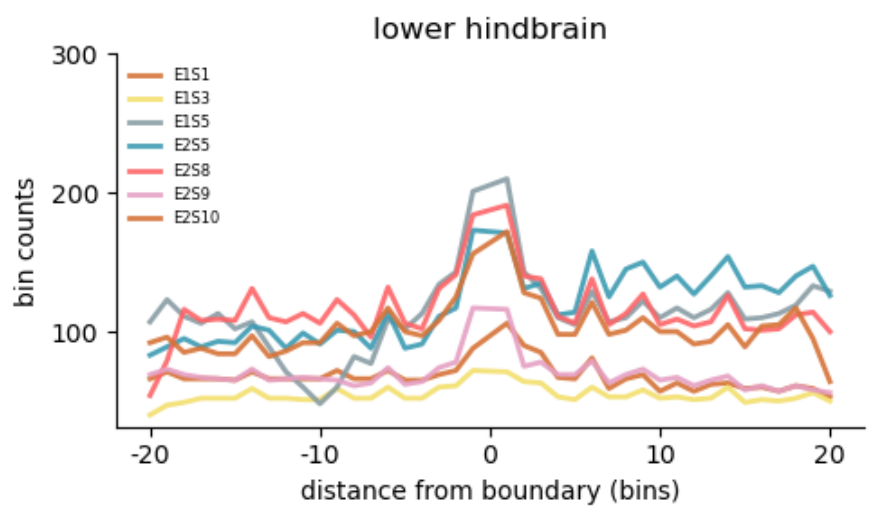

Bin counts at the boundaries of the following regions with the meninges, related to Fig. 2A

(A) Subpallium – meninges. (B) midbrain – meninges. (C) dorsal pallium – meninges. (D) upper hindbrain – meninges (E) lower hindbrain - meninges

Supplementary Figure 2

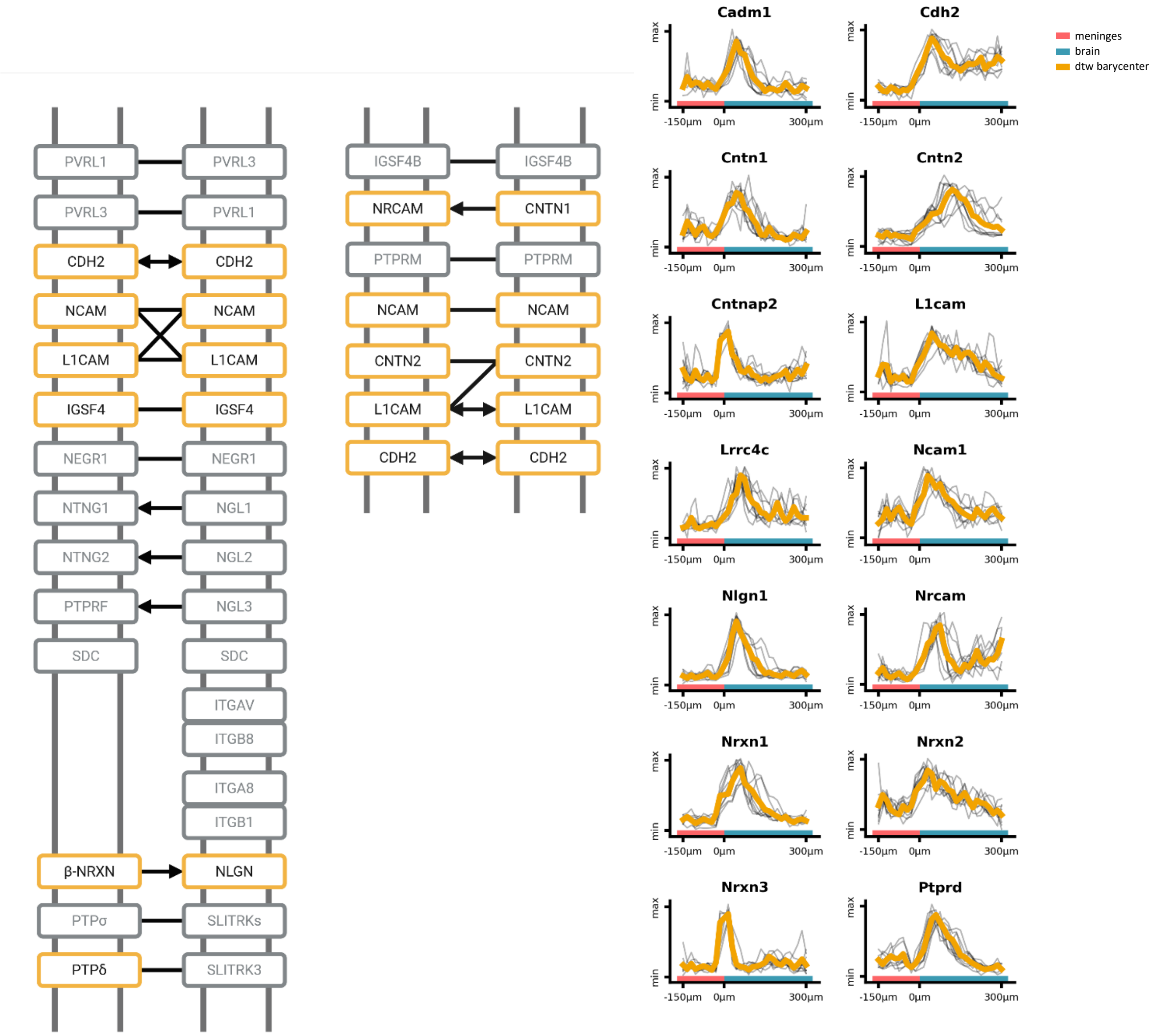

Overview of the selected cell adhesion molecules (KEGG ID: mmu04514) pathway interactions, related to Fig. 4C

Gene expression levels are presented as z-score–transformed mean expression values.

Supplementary Figure 3

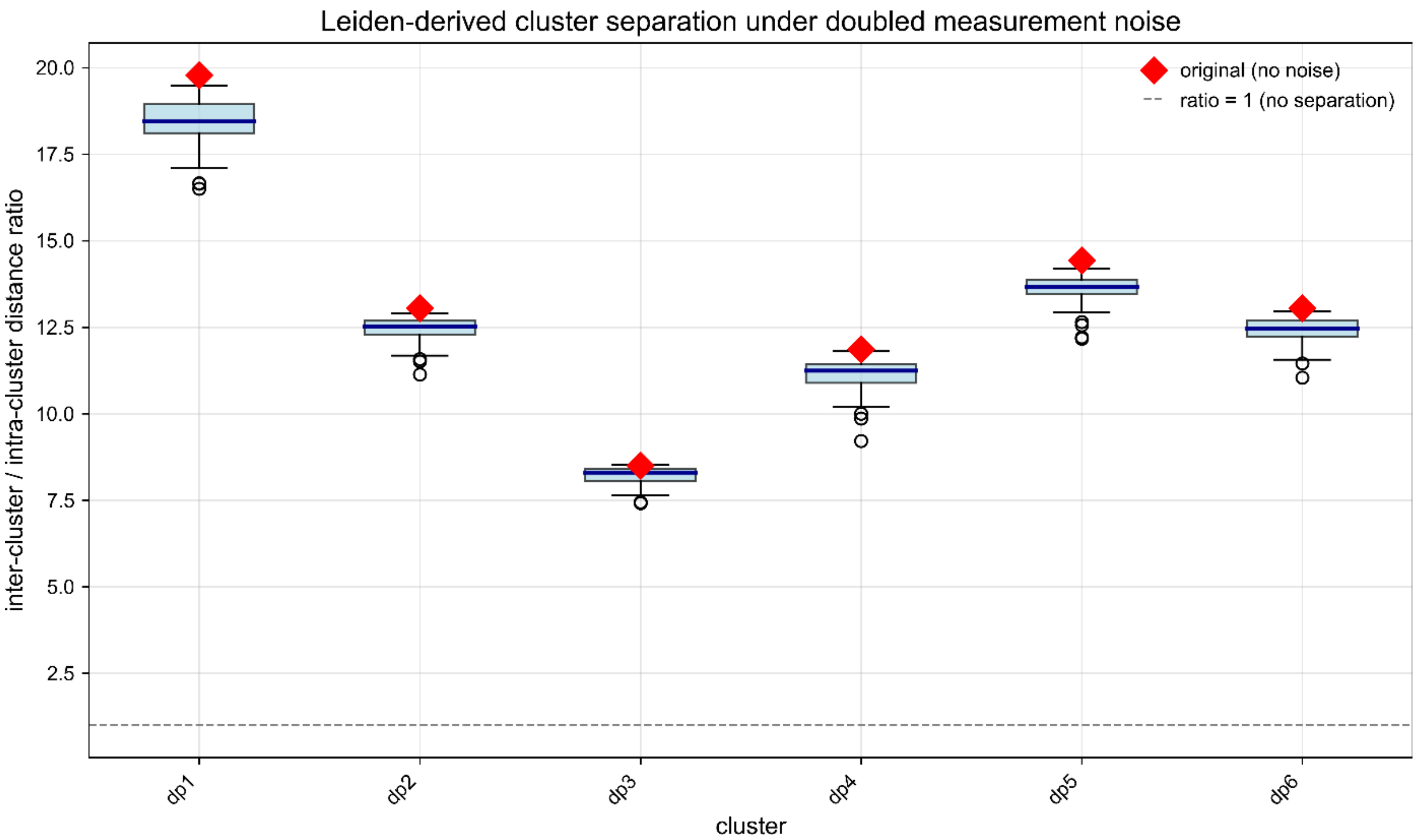

Leiden-derived cluster robustness analysis, related to Fig. 3A

Comparison of intra- and inter-cluster distances of Leiden clustering-derived gene expression patterns, after 1-fold increase of noise levels.

Boxes represent the interquartile range (25th–75th percentiles), center lines indicate medians, and whiskers extend to the most extreme data points within 1.5 × IQR of the lower and upper quartiles. Points beyond the whiskers indicate outliers.

Supplementary Figure 4

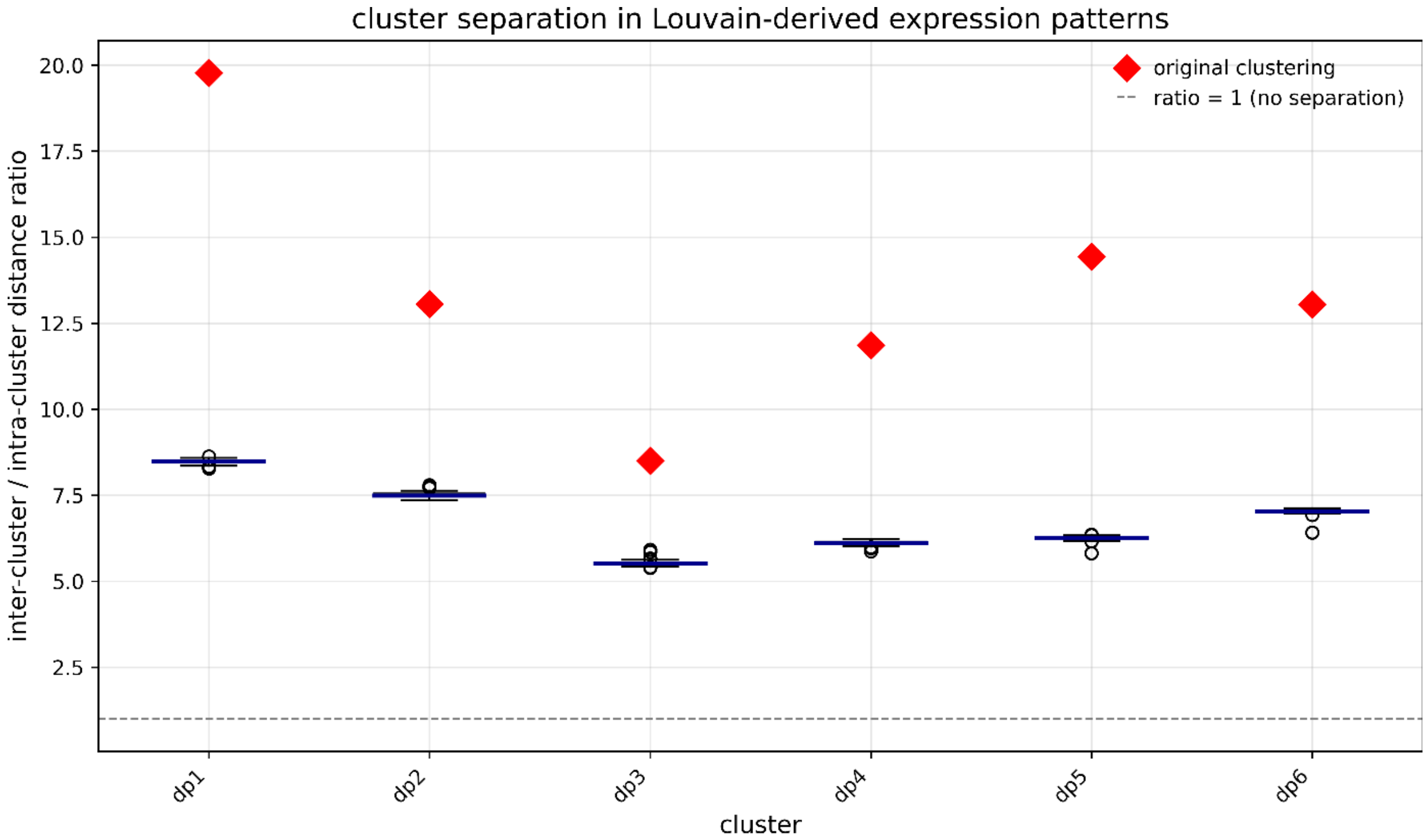

Louvain-derived cluster robustness analysis, related to Fig. 3A

Comparison of intra- and inter-cluster distances of Louvain clustering-derived gene expression patterns. Labels were transferred from the Leiden clustering-derived gene expression patterns clustering.

Boxes represent the interquartile range (25th–75th percentiles), center lines indicate medians, and whiskers extend to the most extreme data points within 1.5 × IQR of the lower and upper quartiles. Points beyond the whiskers indicate outliers.

Supplementary Figure 5

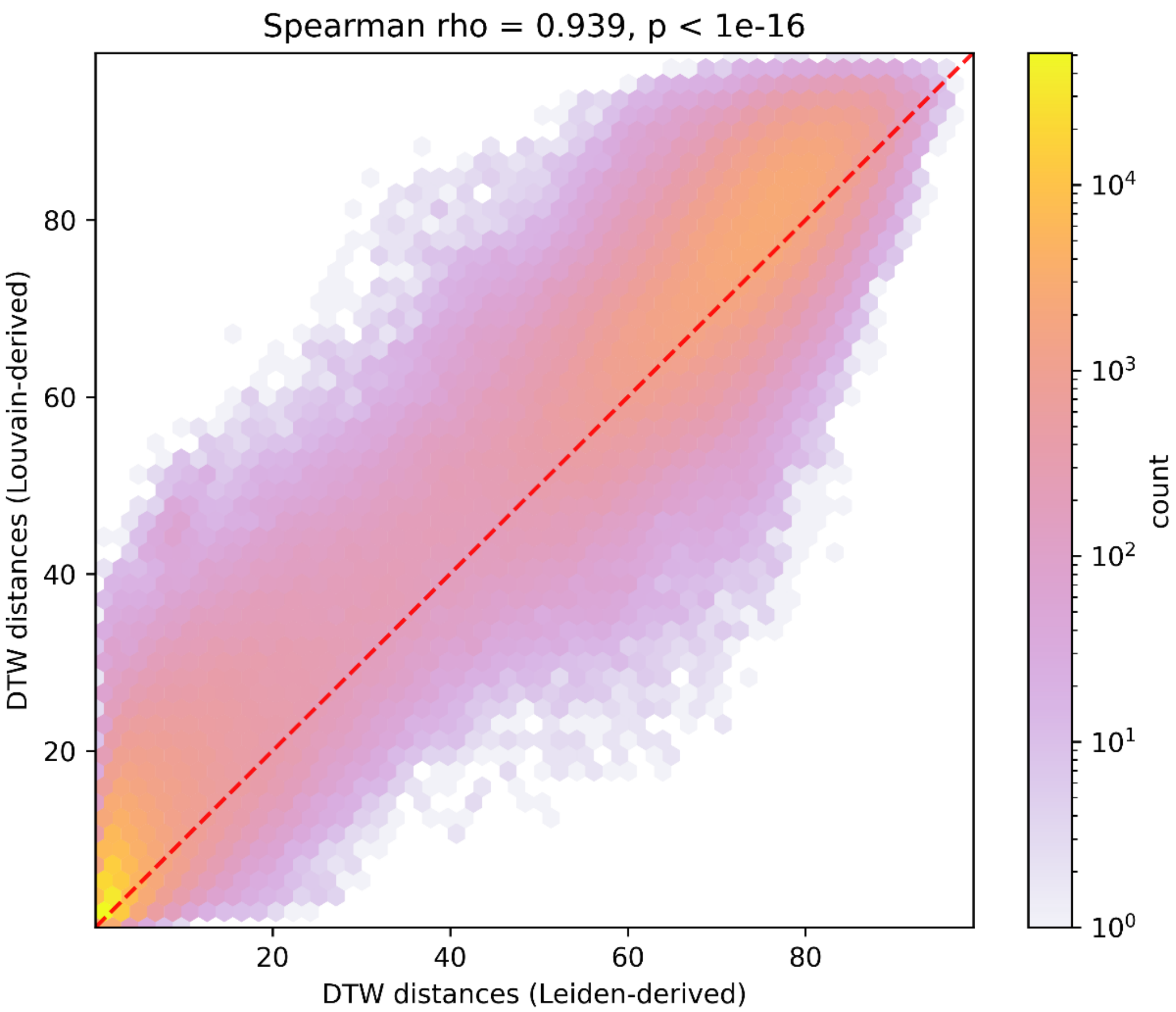

Spearman correlation of Leiden and Louvain based cluster detection, related to Fig. 3A

Spearman correlation analysis of the pairwise DTW distances in Leiden and Louvain-derived gene expression patterns.

Supplementary Figure 6

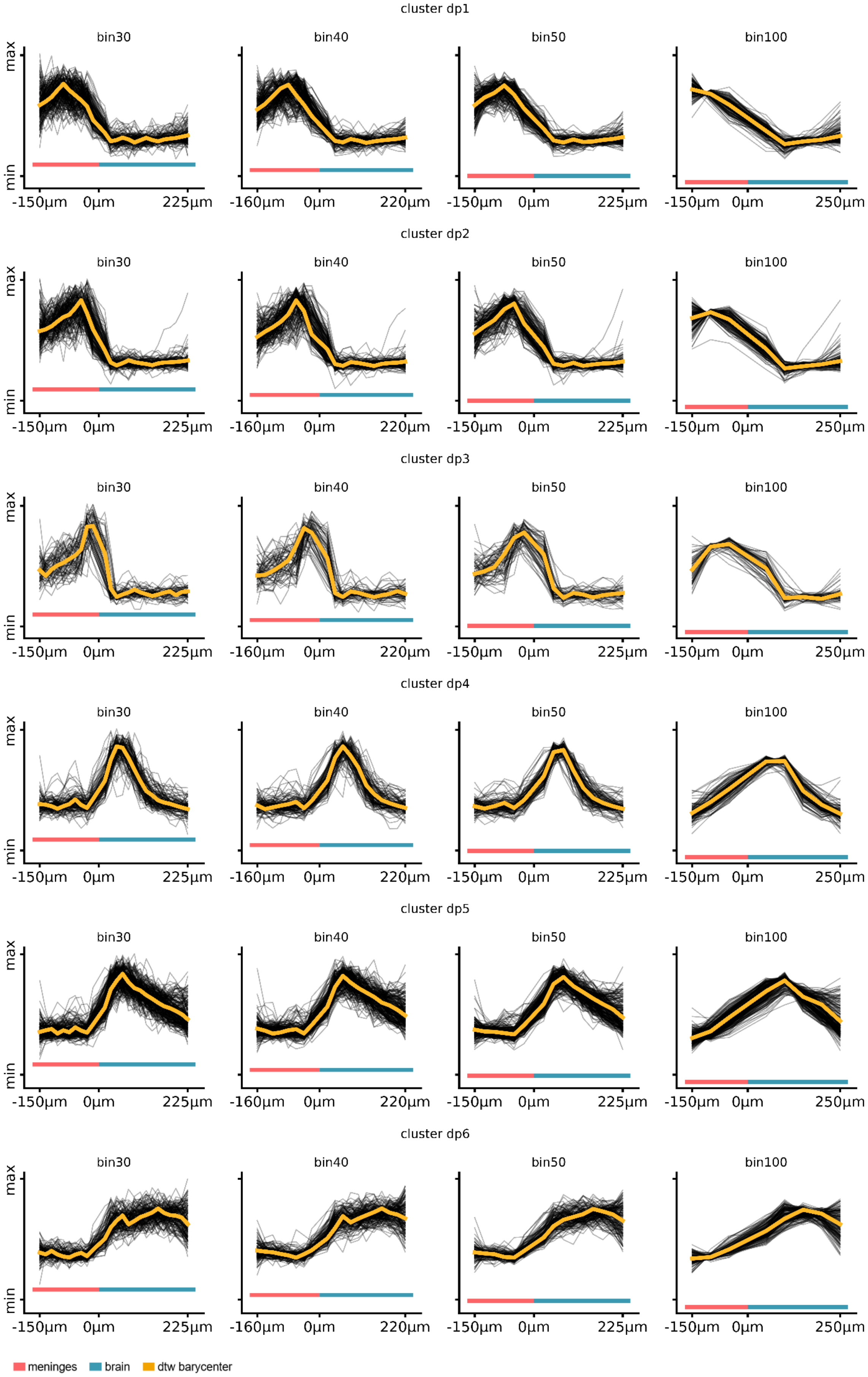

Visualization of the impact of different binning strategies on gene expression pattern resolution, related to Fig. 3A

Cluster expression levels are presented as z-score–transformed mean expression values.

Supplementary Figure 7

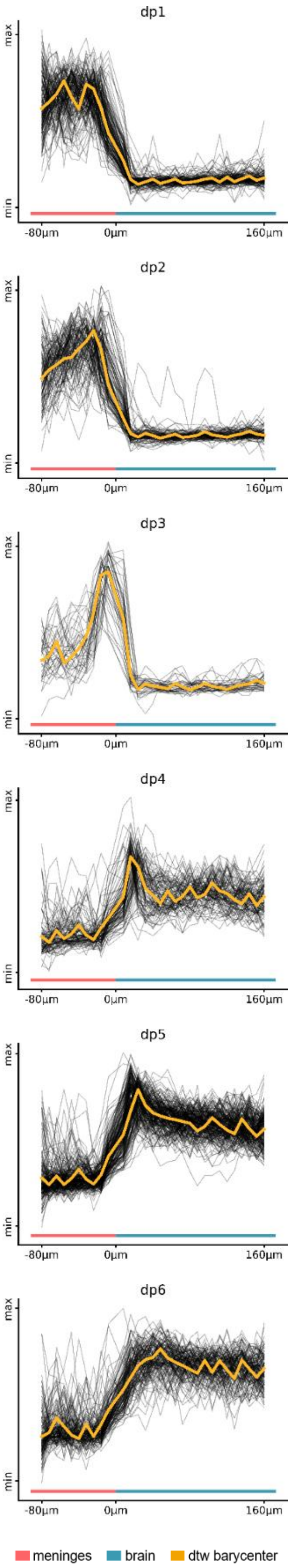

Clusters dp1 to dp6 in E15 and E15.5 VisiumHD datasets, related to Fig. 3A

Cluster expression levels are presented as z-score–transformed mean expression values.

Supplementary Table S1: number of genes in each cluster and boundary region, related to Fig. 2B

	wb1	wb2	wb3	wb4	wb5	wb6
dorpal_men	196	180	222	51	281	145
colsup_men	268	442	15	26	178	256
highhind_men	24	15	3	2	25	15
dorpal_men	196	180	222	51	281	145
subpal_men	68	344	6	6	66	103
lowhind_men	45	98	3	9	38	25

Supplemental Methods

Supplementary Method S1: Assessing robustness of Leiden-derived gene expression pattern clustering

Cluster separation was quantified using the ratio of inter-cluster to intra-cluster dynamic time warping (DTW) distances, where higher values indicate stronger separation between clusters relative to their internal variability. Pairwise DTW distances were computed between gene expression profiles, and cluster-specific ratios were derived by comparing average distances within clusters to those between clusters. To assess robustness, gene expression profiles were perturbed by adding noise estimated from replicate measurements, capturing realistic variation in signal shape and amplitude. To simulate poor Leiden clustering repeatability, we increased the noise level 1-fold. Noise was modeled using DTW-based alignment to derive residual patterns, which were then resampled and added to the mean profiles. Separation ratios were computed for both the original data and across multiple noise-perturbed resamples, yielding per-cluster distributions. This approach enabled evaluation of whether clusters maintain favorable inter- versus intra-cluster distance ratios under realistic noise conditions.

Supplementary Method S2: Assessing robustness of Leiden-derived gene expression pattern clustering

Cluster stability across clustering strategies was further assessed by comparing separation ratios between Leiden- and Louvain-derived expression profiles without additional noise perturbation. Cluster labels obtained from Leiden-based signal clustering were applied to the corresponding Louvain-derived signals, enabling direct comparison of cluster structure across methods. Separation ratios, defined as the ratio of inter-cluster to intra-cluster DTW distances, were computed for the original Leiden signals and for multiple Louvain-derived signal realizations. This yielded per-cluster distributions of separation ratios reflecting variability across Louvain instances. Consistency between the Leiden reference ratios and the Louvain-derived distributions was interpreted as evidence of stable and transferable cluster structure.

Supplementary Method S3: Assessing robustness to upstream clustering with Leiden and Louvain

To compare the impact of the upstream clustering algorithm on the derived time-series patterns, we constructed DTW distance matrices for patterns generated from both Leiden- and Louvain-defined regions. For the Leiden-based analysis, we used the gene expression time series that were originally employed for clustering. For the Louvain-based analysis, we reran the upstream clustering 100 times with different random seeds and, for each gene, computed a DTW barycenter average across its 100 replicate time series. These barycenters were then used as the Louvain-derived gene expression patterns. For each method, we computed the full pairwise Dynamic Time Warping (DTW) distance matrix between all gene-level time series, converted each square matrix to its condensed distance vector, and calculated Spearman’s rank correlation between the Leiden- and Louvain-based DTW distance vectors to assess how similarly the two upstream clustering choices preserve the global pattern similarity structure.

Supplementary Method S4: Selection of Sakoe-Chiba radius for DTW distance calculations

For pairwise DTW distance calculations, we used a larger Sakoe-Chiba radius to accommodate the maximal expected misalignment between two profiles. Because individual gene expression patterns can be shifted by approximately ±1 bin relative to the true boundary, the resulting pairwise misalignment can reach up to 2 bins, which requires a more permissive warping window to ensure valid alignment. In contrast, DTW barycenter averaging aligns each individual profile to the evolving barycenter rather than to every other profile. Under our assumption of small, approximately symmetric shifts across replicates, the barycenter tends to lie between these offsets, so each profile is typically at most ±1 bin away from it. This justifies the use of a stricter Sakoe-Chiba radius of 1 for barycenter averaging, which is sufficient to correct these small shifts while avoiding excessive warping and preserving localized features of the average pattern.
